# Bacterial and Fungal Communities Are Specifically Modulated by the Cocoa Bean Fermentation Method

**DOI:** 10.3390/foods12102024

**Published:** 2023-05-17

**Authors:** Rebecca Ghisolfi, Francesca Bandini, Filippo Vaccari, Gabriele Bellotti, Cristian Bortolini, Vania Patrone, Edoardo Puglisi, Lorenzo Morelli

**Affiliations:** 1 Dipartimento di Scienze e Tecnologie Alimentari per la Sostenibilità della Filiera Agro-Alimentare, Facoltà di Scienze Agrarie Alimentari ed Ambientali, Università Cattolica del Sacro Cuore, Via Emilia Parmense 84, 29122 Piacenza, Italyfrancesca.bandini@unicatt.it (F.B.);; 2 Soremartec srl (Ferrero Group), Piazzale P. Ferrero 1, 12051 Alba, Italy

**Keywords:** microbiota, yeast, lactic acid bacteria, acetic acid bacteria, high-throughput sequencing

## Abstract

Cocoa bean fermentation is carried out in different production areas following various methods. This study aimed to assess how the bacterial and fungal communities were affected by box, ground or jute fermentation methods, using high-throughput sequencing (HTS) of phylogenetic amplicons. Moreover, an evaluation of the preferable fermentation method was carried out based on the microbial dynamics observed. Box fermentation resulted in higher bacterial species diversity, while beans processed on the ground had a wider fungal community. *Lactobacillus fermentum* and *Pichia kudriavzevii* were observed in all three fermentation methods studied. Moreover, *Acetobacter tropicalis* dominated box fermentation and *Pseudomonas fluorescens* abounded in ground-fermented samples. *Hanseniaspora opuntiae* was the most important yeast in jute and box, while *Saccharomyces cerevisiae* prevailed in the box and ground fermentation. PICRUST analysis was performed to identify potential interesting pathways. In conclusion, there were noticeable differences between the three different fermentation methods. Due to its limited microbial diversity and the presence of microorganisms that guarantee good fermentation, the box method was found to be preferable. Moreover, the present study allowed us to thoroughly explore the microbiota of differently treated cocoa beans and to better understand the technological processes useful to obtain a standardized end-product.

## 1. Introduction

Cocoa beans are the raw materials for chocolate production, and their fermentation is still a spontaneous maturing process, usually taking place in the farms where the plantations are located [1]. The different post-harvest practices result in different qualities of the dry fermented final product, driving the economic value of cocoa beans [2,3,4]. The fermentation of cocoa beans mainly involves three microbial groups: yeasts, lactic acid bacteria (LAB) and acetic acid bacteria (AAB), which cooperate in the production of well-fermented cocoa beans. The fermentation activities are exothermic reactions, which provoke an increase in the temperature of the cocoa mass from 25–30 °C up to 45–50 °C, resulting in a decline in all microorganisms [1,3,4].

Yeasts dominate the first 24–48 h of fermentation in limited oxygen availability, acid conditions and high carbohydrate concentrations from the mucilaginous pulp [5].

Between 24 and 72 h, the pulp begins to dry and the amount of air that interacts with the mass increases, thus creating the ideal conditions for the growth of LAB and AAB consecutively [1,5]. During the next 72–112 h, the ethanol produced by yeasts and the lactic acid produced by LAB are oxidized by AAB in acetic acid and acetic acid and acetoin, respectively, with a slight decrease in the cocoa pulp pH. Subsequently, acetic acid is hyperoxidized into carbon dioxide and water [6]. The method and duration of fermentation depend, respectively, on the production area and on the variety of cocoa [3].

Heap fermentation is the simplest and the most common method in the small farms of West Africa. Beans are piled up on a banana-leaf layer, covered with banana leaves and jute bags (to help maintain the heat inside). This is a typical anaerobic-condition fermentation, so after a rest phase of 24–48 h, the beans are mixed and turned over to allow oxygen diffusion [7]. On the other hand, box fermentation, generally wooden boxes, is the most common method around the world, in particular in larger farms of Cote d’Ivoire [8]. The thickness of the box is important to avoid heat loss. Beans are turned by hand for quantities of 25–100 kg while, for larger quantities, they are transferred to successive boxes, arranged in cascade, by fall. Boxes have a holed bottom, which allows the correct drainage of the fermented mass and helps in aeration [7]. Beans can also be fermented in jute bags, closed and covered with banana leaves or plastic sheets to avoid unnecessary dispersion of moisture and heat. Jute bag fermentation is widely used in Ecuador, even if high risks of poor aeration and high humidity could damage the beans irreparably. It is a shorter fermentation, from 67 to 72 h, that requires a higher drying temperature [9].

Two key points of fermentation are juice-pulp drainage and avoiding over-dissipation of heat and moisture. Insufficient pulp drainage will result in bad fermentation and mold contamination, while jute bags or a combination between jute bags and banana leaves is recommended to avoid dissipation of heat and moisture and therefore promote the growth of microorganisms. Choosing the correct method in fermentation significantly impacted process parameters, such as temperature, which influenced the microbial population [9]. Adequate coverage prevents excessive air penetration and unnecessary environmental contamination [8,9].

Several studies investigated microbial populations and dynamics in different fermentation methods, using a culture-dependent approach, an independent approach or both. Nielsen et al. (2007) [10] investigated two methods (tray and heap), while wider microbial analyses were conducted in some studies considering only one fermentation method or a combination of two (box and heap mainly). For example, bacterial species diversity was studied in Brazilian box fermentation by Papalexandratou et al. (2011) [11], both culture-dependent and independent techniques were used to target the bacterial population in heap fermentation [12], and yeast diversity was studied through a culture-independent approach [13]. In addition, heap and box fermentation were compared, for example, by Meersman et al. (2013) for Malaysian cocoa beans [14], Visintin et al. (2016) for West African cocoa beans [15] and Papalexandratou et al. (2011) for Ivory Coast and Brazil cocoa beans [16] using a culture-independent approach. As far as we know, the microbiota of jute fermentation is poorly investigated, and no one has compared this method with the ground and box methods using cocoa from the same origin and farm.

The main purpose of this work was to assess bacterial and fungal diversities according to the different fermentation methods using a culture-independent approach. The three fermentation methods examined in this paper were as follows: (i) in heaps covered with banana leaves (ground); (ii) in wooden or plastic boxes (box); and (iii) in jute bags (jute).

Furthermore, the microbial dynamics of the three methods were evaluated while considering different fermentation times. Subsequently, the focus was to identify by culture-independent analyses the species that are distinctive of the three methods considered and evaluate the preferable fermentation method based on the microbial dynamics observed.

## 2. Materials and Methods

### 2.1. Fermentation Process and Sample Collection

The fermented cocoa beans were collected from a production site in Cameroon during the mid-crop season (June to July 2014). The spontaneous fermentation process was conducted with three different methods: (i) wooden box fermentation (BF); (ii) jute sack fermentation (JF); (iii) ground fermentation (GF).

In this study, the fermentation step lasted six days, and the sampling was carried out in triplicates during each day. The total number of samples obtained and submitted to the next DNA extraction step was 54.

### 2.2. DNA Extraction

DNA extraction was performed according to a previous study [17]. One hundred grams of processed cocoa beans was ground and homogenized, and then Fast DNA Spin Kit for Soil (MP Biomedicals, Solon, OH, USA) was used to collect DNA into sterile tubes. For each sample, DNA was eluted in 100 μL of sterile TE buffer (10 mM Tris/HCl, 1 mM EDTA pH 8.0) and stored at −20 °C. The quantification of the DNA extracted was performed with a Quant-iT HS ds-DNA assay kit (Invitrogen, Paisley, UK) and a QuBit fluorometer. To verify the quality of the DNA sample collected, 2 μL was analyzed by electrophoresis on 0.8% agarose gel.

### 2.3. DNA Amplification

Bacterial and fungal communities were determined by analysis based on HTS of 16S rDNA and the Internal Transcribed Spacer 1 (ITS1) region of ribosomal RNA (rRNA) amplicons.

The amplification of the V3-V4 region of the 16S rRNA gene was carried out by PCR using the universal primers 343f (5′-TACGGRAGGCAGCAG-3′) and 802r (5′-TACNVGGGTWTCTAATCC-3′). The PCR reaction mix was composed of 12.5 μL of Phusion Flash High-Fidelity Master Mix (Thermo Fisher Scientific, Inc., Waltham, MA, USA), 1.25 μL of each primer (10 μM), 0.2 ng of DNA template and nuclease-free water [17]. A second PCR with a forward-tagged primer with a 9-base extension at the 5′ end was applied to perform simultaneous analyses of all the samples in a single sequencing run [18]. The thermocycler program used included the following steps: initial denaturation at 95 °C for 5 min, followed by 20 cycles of denaturation at 95 °C for 30 s, annealing at 50 °C for 30 s, an extension at 72 °C for 30 s, and a final extension at 72 °C for 10 min.

The fungal communities were assessed using the universal primers ITS-1 (5′-TCCGTAGGTGAACCTGCGG-3′) and ITS-2 (5′-GCTGCGTTCTTCATCGATGC-3′) [19]. The PCR reactions required 12.5 µL of Phusion Flash High-Fidelity Master Mix (Thermo Fisher Scientific, Inc., Waltham, MA, USA), 1.25 µL of each primer (10 µM), 2 µL of DNA and nuclease-free water to reach the final volume of 25 µL. The thermocycler was set as follows: an initial hold at 94 °C for 4 min, followed by 28 cycles of 94 °C for 30 s, annealing at 56 °C for 30 s, an extension at 72 °C for 1 min, and a final extension at 72 °C for 7 min.

Two-step PCR is required to reduce the generation of anomalous data caused by the non-specific primer annealing [20]. Amplicons were combined in equimolar ratios and multiplexed into two separate pools, one for Bacteria and the other for Fungi, using the same molecular weights (20 ng). Pools were purified by solid-phase reversible immobilization (SPRI) method using an Agencourt AMPure XP kit (REF A63880, Beckman Coulter, Milan, Italy). The sequencing analysis was carried out by Fasteris S.A. (Geneva, Switzerland) using a TruSeq DNA sample preparation kit (REF 15026486, Illumina Inc., San Diego, CA, USA) for amplicon library preparation. The instrument used for sequencing was MiSeq Illumina (Illumina Inc., San Diego, CA, USA) with V3 chemistry generating 300 bp paired-end reads.

### 2.4. Sequence Data Preparation

Filtering, multiplexing and preparation of data were carried out as previously described [17,21,22], before statistical analyses. Base calling was performed using MiSeq Control Software version 2.3.0.3, RTA v1.18.42.0 and CASAVA v1.8.2, while the “pandaseq” script was used to align raw Illumina sequences [23].

Since V3–V4, 16S rRNA and ITS1 gene amplicons are shorter than 500 bp and require 300 bp paired-end reads per amplicon for reconstructing full regions, a minimum overlap of 30 bp between read pairs and two maximum allowed mismatches were set. To demultiplex the sequences, Fastx-toolkit was applied according to sample indexes and primers. Among the bacterial sequences, homopolymers >10 bp (chimeras), sequences outside the regions of interest and non-targeted taxa were removed [17]. On the other hand, among the ITS amplicons, the UCHIME algorithm with UNITE database v6 was used to identify and discard homopolymers >10 bp, chimeras and non-fungal sequences. Operational taxonomic units (OTUs) were determined and taxonomy-based approaches were performed on the sequences. For the OTUs, Mothur V1.32.1 and taxonomy matrixes for the V3–V4 regions were applied [24]. Mothur was used to perform ITS taxonomy-based analyses with a minimum length of 120 bp and no upper length limit due to ITS variability. Statistical analyses were performed with R2 supplemented with the Vegan package [25]. To visualize the hierarchical clustering of the sequences, the average linkage algorithm was applied at different taxonomic levels. Principal component analysis (PCA), which data did not show, was used to assess the unconstrained sample grouping and the canonical correspondence analysis (CCA) to visualize the significance of different treatments on the analyzed diversity (constrained variance). To estimate the associated α and β diversity, the OTU- and taxonomy-based matrixes were analyzed using R. Moreover, to better estimate any significant differences between samples, α-diversity analyses based on observed richness (S) and Simpson’s diversity index (D) were performed on bacterial and fungal communities. The identification of the most abundant OTUs was confirmed with RDP (Ribosomal Database Project) for Bacteria and with MYCOBANK Database for Fungi.

### 2.5. PICRUST Analysis

To predict the supposed metagenomic profile, based on OTU abundance, Phylogenetic Investigation of Communities by Reconstruction of Unobserved States (PICRUSt2 https://github.com/picrust/picrust2; Version 2.5.1, accessed on 1 February 2023) was applied after the processing of amplicon sequencing analysis of the sample. In PICRUST2, specific 16S-seq abundances were used to deduce the abundances of enzymes and metabolic pathways for each sample [26]. The two databases used to analyze the PICRUST results were the Kyoto Encyclopedia of Genes and Genomes database (KEGG; http://www.genome.jp/kegg/, accessed on 15 February 2023) and Statistical Analyzer of Metagenomic Profiles (STAMP 2.3.1: https://beikolab.cs.dal.ca/software/STAMP, accessed on 1 March 2023). MetaCyc [27] was used, in the end, to elaborate outputs.

## 3. Results and Discussion

### 3.1. Overview of the Bacterial and Fungal Communities Involved in Different Fermentation Methods

Sequencing of bacterial amplicons resulted in 1200 high-quality sequences per sample after screening, filtering and rarefaction. The dataset revealed a consistent coverage of 93.83%, and sequences were classified to family level (99.6%), genus level (89.5%) and species level (9.3%) to a lower extent. For fungal communities, a total of 12,351 high-quality sequences per sample were obtained, and the coverage rate was 99.54%. The sequences were classified to family level (65.3%), genus level (55.1%) and species level (43.0%).

**Figure 1:**
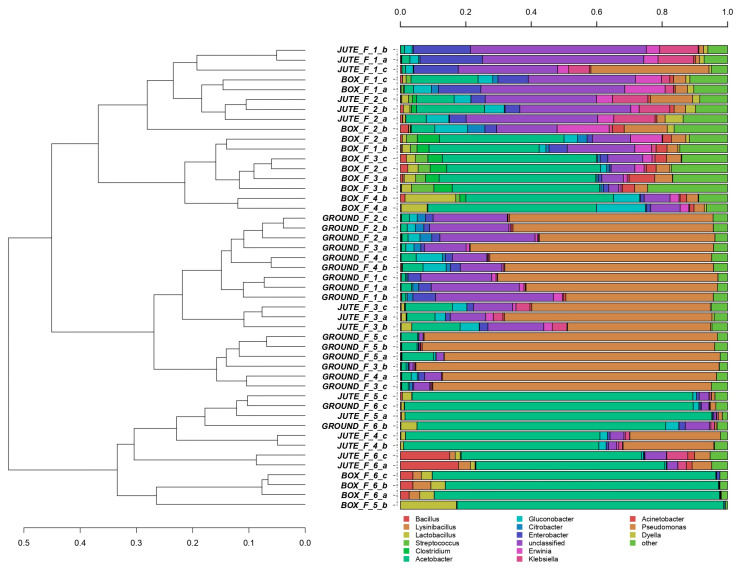
Hierarchical clustering of bacterial sequences using the average linkage algorithm at the genus level for taxa with at least ≥5% of participation in a single sample. Taxa beneath this threshold were added to the sequence group named “other”. Image legend: method; “F_n”, days of fermentation; “a, b, c”, replicates 1, 2 and 3.

A comprehensive picture of the most abundant bacterial genera is shown in Figure 1, confirming differences between the three investigated methods and fermentation days. The samples are quite well clustered, even if some similarities are present.

Two main clusters were identified, according to the days of fermentation. The first group contains samples from the three fermentation methods (JF, jute fermentation; GF, ground fermentation; BF, box fermentation) from day 1 up to day 4; in the second group, samples from the last two days of fermentation (5 and 6) were mostly represented.

More specifically, three sub-groups were identified in the first cluster, corresponding mainly to the three fermentation methods studied. The first one included day 1 of BF and days 1 and 2 of JF displaying the prevalence of *Enterobacter* and *Enterobacteriaceae* non-classifiable at a deeper taxonomical level. The second sub-group was dominated by the *Acetobacter* genus in BF at days 2, 3 and 4 of fermentation. The third, well-defined group included samples from days 1 to 5 of the GF and day 3 of the JF method. From day 1 to 5 of GF and on day 3 of JF, the dominance of the *Pseudomonas* genus together with *Enterobacteriaceae*_unclassified was observed.

The second cluster included all samples from the last days of the three fermentation methods analyzed. *Acetobacter* was the dominant genus in all the samples, but with some exceptions: (I) *Pseudomonas* genus was detected on day 4 of JF and disappeared in the next two days; (II) in the last day of JF and BF, *Bacillus* genus was observed; (III) the presence of ex-*Lactobacillus* genus was mainly found in BF method, but also at the end of JF and GF. *Lactobacillus fermentum* was one of the main LAB species isolated from BF or GF [12,28,29], with *Leuconostoc pseudomesenteroides* (glucose and fructose fermenting), isolated only at the beginning of GF [12]. *L. plantarum*, detected in all the fermentation processes according to [29], and *L. fermentum* were the most prevalent LAB species dominating both in BF and in GF [10,12,15,30]. More specifically, *L. plantarum* strains were observed to be abundant at the beginning of the GF, while *L. fermentum* strains converted fructose into mannitol during longer fermentation time [12]. Citrate-converting LAB species, such as *L. fermentum*, play a role in pH increasing, which is important to promote AAB growth and the pectinolytic activity of yeasts [28]. Common precursors of aromatic amino acids and secondary metabolites in the shikimate pathway were identified in *L. fermentum* and other species, not observed in this study [31].

In agreement with a work conducted in 2013 to identify a core and variable microbiota in the box and the heap cocoa bean fermentation [14], *Lactiplantibacillus plantarum* (formerly *Lactobacillus plantarum*) and the AAB *Acetobacter pasteurianus* emerged as the core bacterial population.

*Gluconobacter* and *Acetobacter* genera were observed at the beginning and during the BF, respectively. Similar results for *Gluconobacter* sp. and *Acetobacter pasteurianus* were found in the literature for Ecuadorian and Ivory Coast cocoa BF [5,28,29]. Moreover, the *Gluconobacter* genus was also found in the JF and the GF method on days 2, 3 and 4. This microorganism was reported in the literature as one of the most abundant acetic acid bacteria during heap fermentation, thanks to the turning and mixing of beans [31].

Among the lactic acid bacteria involved in spontaneous cocoa bean fermentation, the ex-*Lactobacillus* genus is one of the most relevant for its flavor precursors, citrate converting and mannitol production [12,28]. In this study, it was mainly detected in BF throughout the process, especially in the first four days. Lactobacilli were also found in the JF method. Visintin et al. (2016) [15] isolated different species of *Lactobacillus* during different days of GF. These results were in contrast with this study, in which *Lactobacillus* was observed in low abundance on the last day of the GF.

In summary, at the beginning of all the fermentation processes, the *Acetobacter* genus dominated mainly in the BF, and the *Pseudomonas* genus dominated mainly in the GF samples. Spoilage microorganisms could be found at the beginning of GF and BF [12,29]; examples include some enterobacterial species, which may convert glucose into lactic acid and citric acid [12]. Different studies reported *Enterobacteriaceae* during the initial phase of cocoa bean fermentation [28], but in this work, a few representatives could be found at the end of both GF and JF.

**Figure 2:**
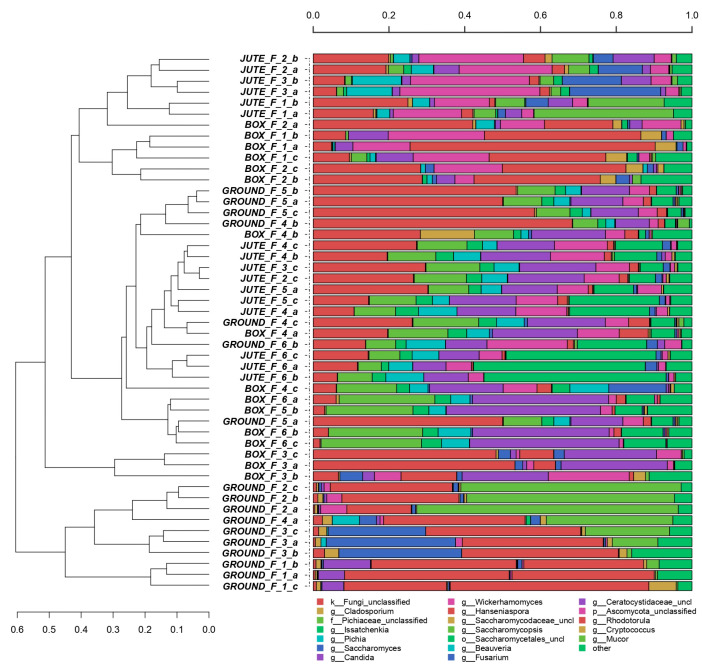
Hierarchical clustering of fungal sequences using the average linkage algorithm at the genus level for taxa with at least ≥5% of participation in a single sample. Taxa with low contributions were added to the “other” group. Image legend: “Fermentation_n”, days of fermentation; “a, b, c”, replicates 1, 2 and 3.

A comprehensive picture of the most abundant yeast genera is shown in Figure 2. The samples are less clustered than bacterial genera and present, in some cases, an abundance of unclassified fungi.

On days 1 and 2 of the BF and from days 1 to 3 of the JF, the three most relevant genera observed were *HanseniaspIora*, *Pichia*, *Wickerhamomyces* and *Saccharomycopsis*. *Hanseniaspora* and *Rodotorula* were the dominant genera on day 1 of the GF, with a slow decrease over the process. As reported in [28], species of *Hanseniaspora* represented a minority during cocoa fermentation and were detected mainly at the beginning of the spontaneous process. This study observed a clear dominance of other genera in the BF, GF or JF over time. In another study, *Hanseniaspora opuntiae* predominated during the whole wooden BF, with different species of *Candida* and *Pichia* according to the days of fermentation [29].

*Pichiaceae* and *Saccharomycetaceae* families and *Candida* and *Pichia* genera were the dominant yeasts at the end of the BF. These results were in accordance with microorganisms isolated from cocoa beans fermented in West Africa [15].

A coexistence of *Saccharomyces*, *Hanseniaspora, Pichia*, *Candida* and *Wickerhamomyces* genera was observed from day 3 to the end of the GF method. According to the literature, isolates of *Saccharomyces* sp., *Candida* sp., two species of *Hanseniaspora*, *Pichia* sp. and *Schizosaccharomyces* sp. were also involved in the ground cocoa bean fermentation in West Africa, without a specific dominant yeast [15].

In the JF method, *Pichia*, *Candida* and *Wickerhamomyces* genera and the *Saccharomycetaceae* family were identified. More precisely, from day 4 to day 6, the *Saccharomycetales* family grew steadily, becoming dominant at the end of the JF. *Candida* sp., mainly detected in the middle and at the end of the process, could be a specific marker of incomplete fermentation [29].

### 3.2. Effects of the Method and the Days of Fermentation on the Abundance of OTUs

**Figure 3:**
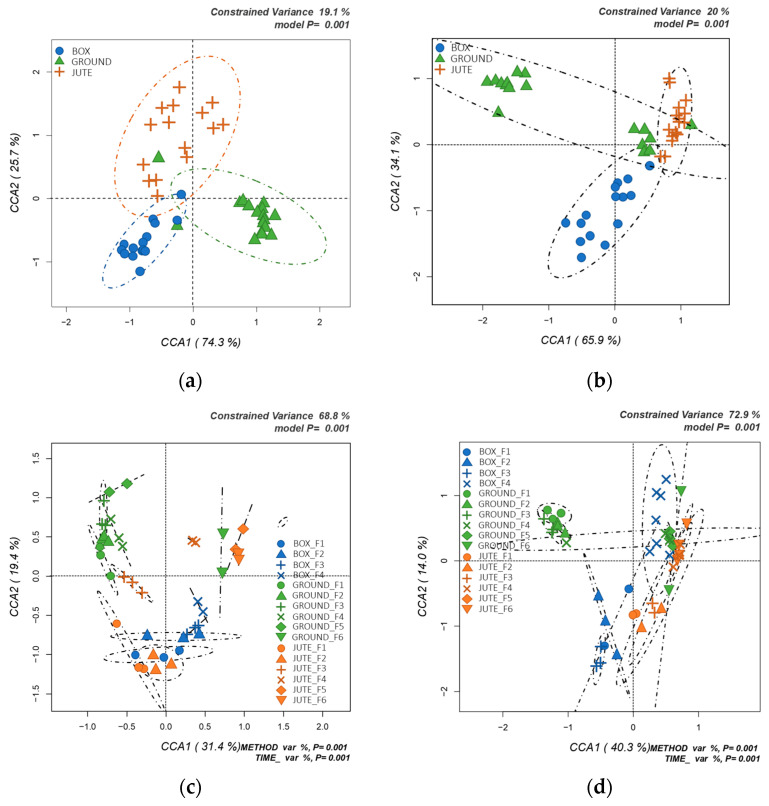
(**a**) db_RDA (distance-based redundancy analyses) to test the influence of the fermentation method on the bacterial OTUs in cocoa beans. (**b**) db_RDA (distance-based redundancy analyses) to test the influence of the fermentation method on the yeast OTUs in cocoa beans. (**c**) db_RDA (distance-based redundancy analyses) to test the influence of the fermentation method and the day of fermentation on the bacterial OTUs in cocoa beans. (**d**) db_RDA (distance-based redundancy analyses) to test the influence of the fermentation method and the day of fermentation on the yeast OTUs in cocoa beans.

Total bacterial communities in the cocoa bean fermentation samples were also assessed with an OTU-based approach. Hypothesis-driven, canonical correspondence analysis (CCA) on the relative abundance of the total bacterial OTUs was performed firstly to test the effect of the fermentation method on bacterial ecology (Figure 3a), and then to evaluate the interaction between fermentation method and time (Figure 3c). Moreover, the CCA model was also applied to the total fungal OTUs (Figure 3b,d). All the models were significant (*p* = 0.001), with a quite good explanation of constrained variance (19.1% for Figure 3a, 20% for Figure 3b, 68.8% for Figure 3c and 72.9% for Figure 3d).

Clustering of samples was observed when testing the hypothesis of how the fermentation method influenced microbial dynamics (Figure 3a). GF samples were mainly grouped in the lower right sector of the CCA plot (positive correlation with CCA1 and negative with CCA2), while box samples were in the lower left part (negative correlation with both CCA1 and CCA2). On the other hand, JF samples were highly dispersed in the upper part of CCA2, and they related both positively and negatively with CCA1. In conclusion, the fermentation method influenced the bacterial community; the samples from the different methods appear to be well separated, with ellipses of varying sized intersecting at the center of the model. The ellipse related to JF was wider than the GF ones that were in direct contact with sources of microorganisms, such as soil and air. In contrast, BF showed less variability. Camu et al. (2007) [12] observed limited biodiversity and targeted population dynamics of both lactic and acetic acid bacteria during heap fermentation of Ghana cocoa: *L. plantarum*, *L. fermentum*, *Leuconostoc pseudomesenteroides* and *Enterococcus casseliflavus* dominated among the LAB isolates, while *A. pasteurianus*, *Acetobacter syzygii*-like bacteria, and *Acetobacter tropicalis*-like bacteria were observed among AAB. Furthermore, Papalexandratou et al. (2011) [16] showed the influence of local fermentation practices on species diversity and community dynamics by comparing heap and box fermentation in an Ivorian farm and box fermentations conducted in two different Brazilian farms. It was observed that there was a limited species diversity of lactic and acetic acid bacteria. *L. fermentum* and *Leuconostoc pseudomesenteroides* were the predominant LAB species, but *Erwinia soli* and *Pantoea* sp. specifically dominated both box and heap Ivory Coast fermentations. A wider microbial species diversity was studied in box fermentation of the two Brazilian farms, with a dominance of acetic acid bacteria.

Figure 3b shows the yeast communities analyzed according to the different fermentation methods. The majority of JF and GF samples clearly overlapped and positively correlated with CCA2. On the other hand, the BF method was negatively correlated with CCA2 but equally distributed between positive and negative CCA1. The elongated ellipse of GF samples explained a greater variability compared to the other two methods. The model showed less distinction between different fermentation methods, and specifically, the JF method had less variability. Detailed analysis of the Malaysian microbial population during cocoa fermentation was conducted by Meersman et al. (2013) [14]. They identified a limited number of different LAB species, with *L. fermentum* and *A. pasteurianus* the two dominant species, and a much higher fungal diversity with four dominant species and a large number of yeasts occurring in specific fermentation methods.

The hypothesis studied in Figure 3c is the time–method effect on the bacterial community, which showed lower variability. Samples from days 5 and 6 of BF were missing in this model as a result of filtering steps during the bioinformatics analysis. A positive correlation to CCA2 for GF samples and a negative one for BF samples were represented in the figure. Only the JF samples were clearly clustered by the method and the time: days 1, 2 and 3 were negatively correlated to both CCA1 and CCA2; days 4, 5 and 6 were positively correlated to both CCA1 and CCA2. Similarities were observed between day 5 and 6 of JF and day 6 of GF. GF samples clustered independently of the day of fermentation compared with the other methods and differing only at day 6. In contrast, samples from different days of fermentation in JF and BF were distributed separately within the model.

Figure 3d represents the method and time effects on the fungal community. First, more heterogeneity was generally observed, apart from the GF samples. Days 1, 2, 3 and 4 of GF overlapped and were well separated by all the other samples. All the days of JF were positively correlated with CCA1, but days 1, 2 and 3 were negatively correlated with CCA2, while days 4, 5 and 6 showed a positive correlation. For BF samples, days 1, 2 and 3 overlapped, while day 4 is well differentiated and positively correlated to both CCA1 and CCA2. The fermentation method also seemed to influence the fungal communities involved during different days of fermentation.

### 3.3. Influence of the Fermentation Method on the Bacterial Communities

**Figure 4:**
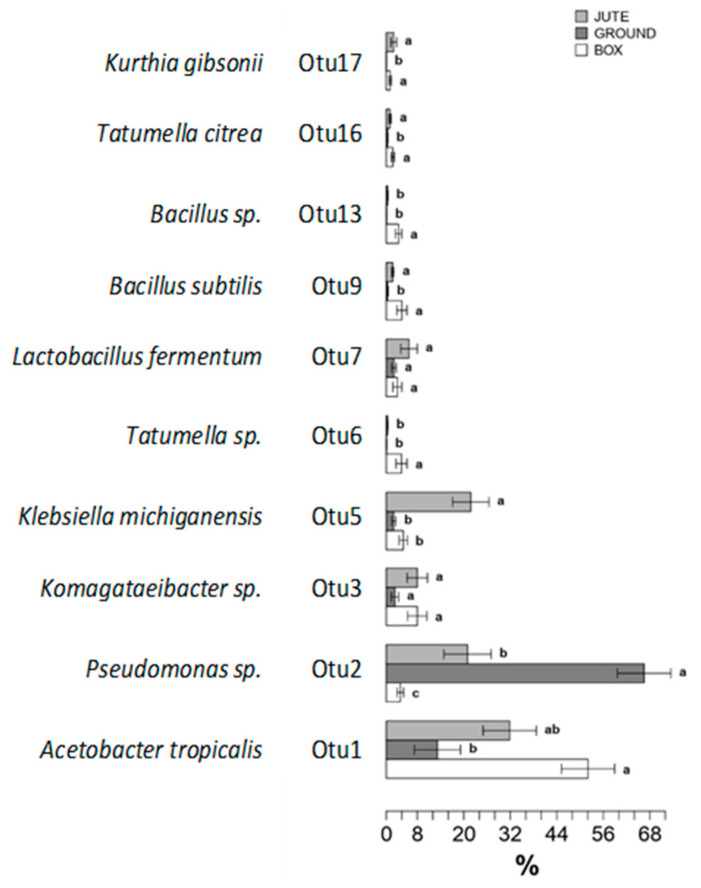
Metastats model to evaluate the effects of the method on the relative abundances of the 10 most abundant OTUs observed comprising 95% of the bacterial diversity in fermented cocoa beans. OTUs showing significant differences according to the false discovery rate correction are highlighted with letters. The assigned species was indicated where possible for each OTU. Significant differences in OTUs are reported due to the false discovery rate correction: bars followed by different letters were statistically different from (a, b, c) or similar to (a, b, ab) each other (Tukey’s test, *p* < 0.05).

The Metastats model (Figure 4), which is an OTU-based approach, was used to better understand the main bacterial species involved in the three different fermentations. The model evaluated the effects of fermentation type on the relative abundances of the 10 most abundant OTUs comprising 95/99.9% of the bacterial diversity in each method analyzed. The taxonomical affiliations of the most abundant OTUs identified were confirmed with Ribosomal Database Project (RDP).

OTU2 (*Pseudomonas* sp.) was the most abundant OTU in GF methods (about 65% in abundance), and it was statistically different from the abundance in the other two methods (32% in JF and less than 8% in BF). This bacterium is associated with soil and water according to Lima Serra et al. (2019) [32].

Differently, OTU13 (*Bacillus* sp.) and OTU6 (*Tatumella* sp.) were present exclusively in BF, at around 8% abundance. *Bacillus* spp. were observed in cocoa bean samples under higher temperatures and less acidic and more aerobic conditions, usually at the end of the process [12]. Numerous *Bacillus* spp. were thermotolerant, but some were isolated after drying and roasting, suggesting that they were able to grow at the highest temperatures [33]. Under fermentative conditions, species of *Bacillus* released different chemical compounds that confer acidity and off-flavors of beans, influencing the final chocolate quality [4,33]. On the other hand, the role of this microorganism and its potential production of valuable flavor and bioactive compounds must be further demonstrated [34,35]. These species produced organic acids as part of their central and secondary metabolism, lactic and acetic acids, pyrazines and 2,3-butanediol, as well as enzymes, such as β-glycosidases, proteases, amylases and lipases [4,32,33,34].

The genus *Tatumella* (OTU16 and OTU6), a member of the *Enterobacteriaceae* family, was statistically present only in BF. Papalexandratou et al. (2011) [28] found two species of *Tatumella* involved in the initial phase (during the first 36 to 42 h) of the Ecuadorian box and platform cocoa bean fermentation. *Tatumella* sp. was also detected in Brazilian box fermentation at 48 h, according to Garcia-Armisen et al. (2010) [36]. *Tatumella* sp. was also observed to produce gluconic acid [28,37]. In the initial phase of cocoa bean fermentation, *Enterobacteriaceae* species might have a role in the pectin degradation of cocoa pulp, converting glucose into lactic and citric acid and gluconic acid production [28,29,37]. Soil could be considered the source of *Enterobacterial* species in cocoa beans, and *Tatumella* sp. is a potential phytopathogen in cocoa pods [36].

OTU5, identified as *Klebsiella michiganensis*, dominated the JF method, for more than 20% abundance, which was statistically different from the other two methods studied. The genus *Klebsiella* represented a minority in cocoa bean fermentation [31].

*Acetobacter tropicalis*, OTU1, was dominant in BF (about 50% abundance), which was not statistically different from the JF method (about 32% abundance) and statistically different from the GF (15% abundance). *Acetobacter* genus is reported in the literature as the main representative acetic acid bacteria, with a particular reference to *Acetobacter pasteurianus* species [12,15,28]. On the other hand, *Acetobacter tropicalis* was observed by Soumahoro et al. (2020) [38] as one of the dominating AAB species in Ivorian heap cocoa fermentation. According to the literature [10,39], *A. tropicalis* remains constant between 24 and 120 h of fermentation, dominating the later stages of fermentation. Different studies isolated *A. tropicalis* from Thailand and Indonesian fruits and flowers [39,40].

### 3.4. Influence of the Fermentation Method on the Fungal Communities

**Figure 5:**
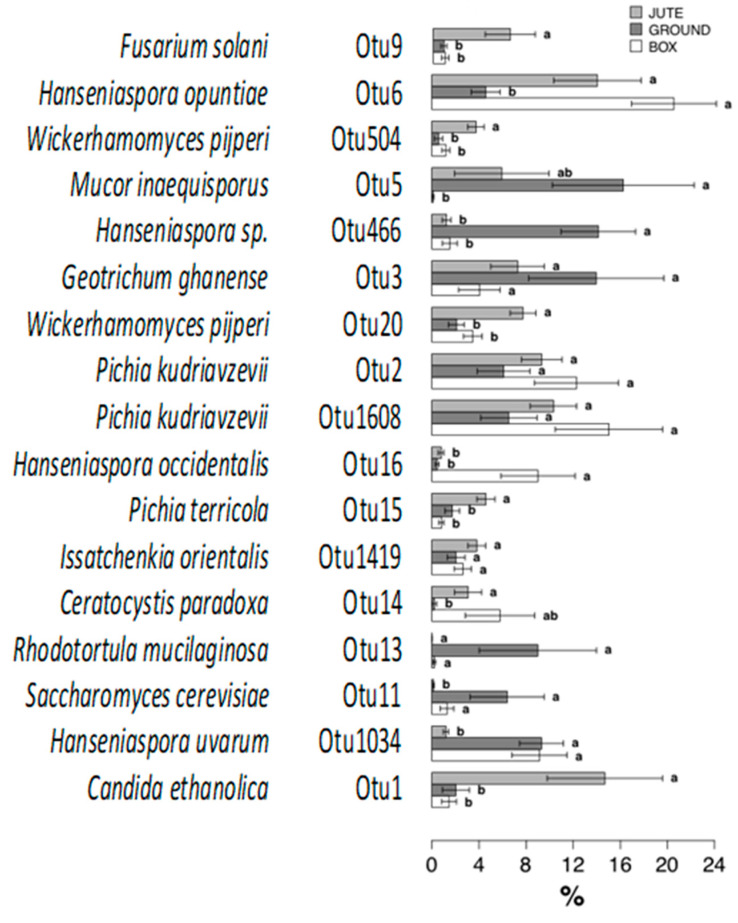
Metastats model to evaluate the effects of the fermentation method on the relative abundances of the 17 most abundant OTUs observed comprising 95% of the fungal diversity in fermented cocoa beans. OTUs showing significant differences according to the false discovery rate correction are highlighted with letters. The assigned species was indicated where possible for each OTU. Significant differences in OTUs are reported due to the false discovery rate correction: bars followed by different letters were statistically different from (a, b) or similar to (a, b, ab) each other (Tukey’s test, *p* < 0.05).

Figure 5 shows the Metastats model for the most abundant OTUs detected during cocoa bean fermentation with different methods. OTU9 (*Fusarium solani*), OTU504 and OTU20 (*Wickerhamomyces pijperi*), OTU15 (*Pichia terricola*) and OTU1 (*Candida ethanolica*) were detected as the most abundant (about 10–15%) in JF and statistically different from GF and BF methods (both less than 4%).

*Candida ethanolica* dominated the last days of BF and the whole process in the GF method [10,15,41]. This study confirmed the observation of the genus *Candida* mainly at the end of the BF and GF methods (Figure 2), but according to OTU abundances, it prevailed in JF (more than 12%). *Candida* and *Pichia* are important yeast genera in cocoa bean fermentation for their metabolization of citric acid, which increases pulp pH and creates an advantageous environment for bacteria [33]. *C. ethanolica*, according to the literature [15], is important for its glycosidase and pectinolytic activities.

No statistically significant differences were observed for OTU3 (*Geotrichum ghanense*), OTU2 and OTU1608 (*Pichia kudriavzevii,* formerly *Issatchenkia orientalis*), OTU1419 (*Issatchenkia orientalis*) and OTU13 (*Rhodotorula mucilaginosa*). Several yeast species, such as *P. kudriavzevii* and *S. cerevisiae*, were identified as actors in cocoa bean fermentation throughout the world [28,42]. On the other hand, as reported in detail by Douglass et al. (2018) [43], the opportunistic pathogen *Candida krusei*, responsible for about 2% of *Candida* species infections in humans, and the important industrial yeast *Pichia kudriavzevii* (regarded as non-pathogenic) are the same species. So, caution and detailed analyses may be needed before the use of *P. kudriavzevii* strains for food application.

*Hanseniaspora opuntiae* (OTU6) was abundant but not statistically different between JF (about 15% abundance) and BF (about 18% abundance). This result was aligned with previous studies [15,28]: *Pichia kudriavzevii* (OTU2 and 1608) and *Hanseniaspora opuntiae* (OTU6) were the main yeast species detected in the BF, but not in GF.

*H. opuntiae* was identified as the unique yeast strain present on fresh cocoa beans. This species usually dominated during cocoa bean fermentation [12,29,44] or participated with other yeast species at the beginning of fermentation [28,45]. This genus is also reported as a less-fermentative yeast, producing ethanol at a concentration lower than that produced by highly fermentative yeasts such as *S. cerevisiae* [45].

On the other hand, OTU466 (*Hanseniaspora* sp.) was statistically different and was observed only for GF (more than 16% abundance) in this study. OTU16 (identified as *Hanseniaspora occidentalis*) was abundant (more than 8%) in BF and statistically different as compared to the other two fermentation methods. *Hanseniaspora uvarum* (OTU1034) was equally observed in both GF and BF methods (more than 8%): according to Visintin et al. (2016) [15], this species was the most isolated at time zero in BF. Some species of *Hanseniaspora*, particularly *H. uvarum*, were identified to be positive for β-glucosidase, glycosidase, pectinase and esterase activity [15]. *Hanseniaspora* spp. were relevant for the impact on final cocoa quality as they produce ethanol, secondary products (organic acids, ketones, esters) and glycosidase [4,37].

*Mucor inaequisporus* (OTU5) was statistically different for GF and BF with about 16% abundance in the samples obtained by the GF method. *Mucor*, specifically *Mucor racemous*, dominated the whole fermentation process studied in [33]. On the contrary, *Ceratocystis paradoxa* (OTU14) was statistically different between GF and JF.

According to the literature [12,15,29,31], *Saccharomyces cerevisiae* is the most abundant species in BF and GF, and it was confirmed in this study with the presence of OTU11 only in BF and in GF methods. In a previous study [15], *S. cerevisiae* was positive for β-glucosidase, glycosidase, protease (influencing) and pectinase activities, having an impact on the final quality [46]. Pectinase activity is important for pulp degradation, whereas protease activity influences first the concentration of amino acids within the beans and then chocolate flavor due to the formation of pyrazines during roasting. The fermentation of sugars in the pulp (β-glucosidase and glycosidase activity) impacted volatile metabolites (e.g., organic acids, esters, aldehydes, ketones) and final aromatic properties [45].

### 3.5. Biodiversity Indexes

The α-diversity analysis was based on observed richness (S) and Simpson’s diversity index (D). The Simpson index quantifies the biodiversity of habitat and is based on the richness and uniformity of species [47]. Good’s coverage was calculated to evaluate the percentage of diversity obtained by sequencing [48]. These are indicators of richness and evenness, and they calculate the value of diversity, a value that increases with the number of species and the measure of equidistribution (uniformity, equality).

**Figure 6:**
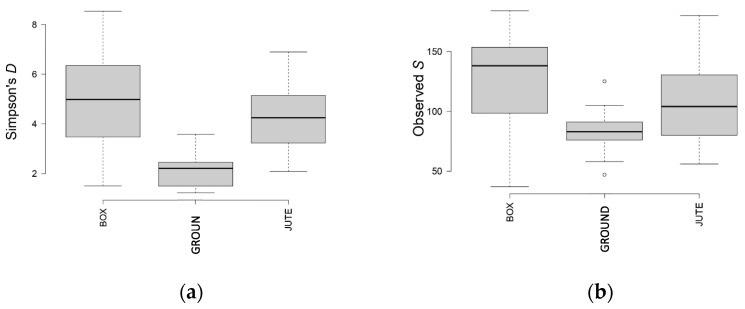
Simpson’s (**a**) and observed (**b**) richness indexes for the analyzed samples. BAC OTUs were classified according to different fermentation methods.

**Figure 7:**
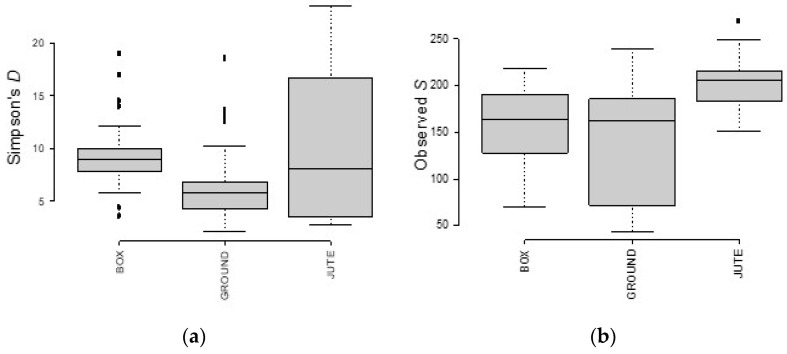
Simpson’s (**a**) and observed (**b**) richness indexes for the analyzed samples. ITS OTUs were classified according to different fermentation methods.

The number of bacterial species in BF and JF samples was high and not equidistributed (Figure 6a), while the number of fungal species was lower for BF and GF compared to JF samples (Figure 7a), from which wider species diversity could be deduced.

As shown by the observed S index in Figure 6b, BF and JF samples had higher variability than GF in terms of bacterial species. On the contrary, the fungal species variability of GF was much higher compared to BF and JF (Figure 7b).

### 3.6. PICRUST Analysis

**Figure 8:**
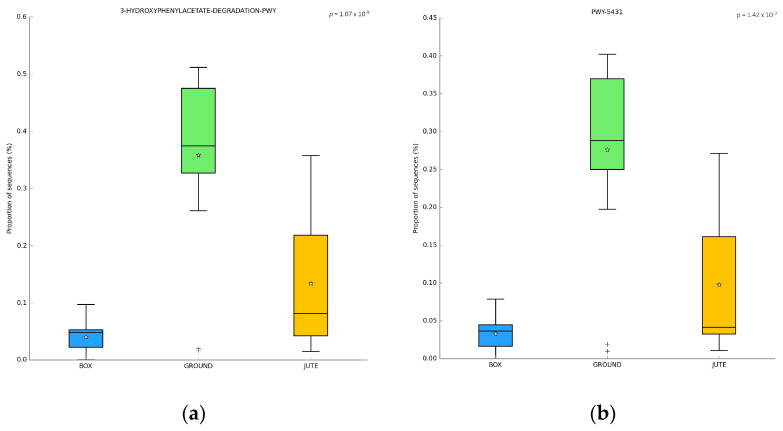

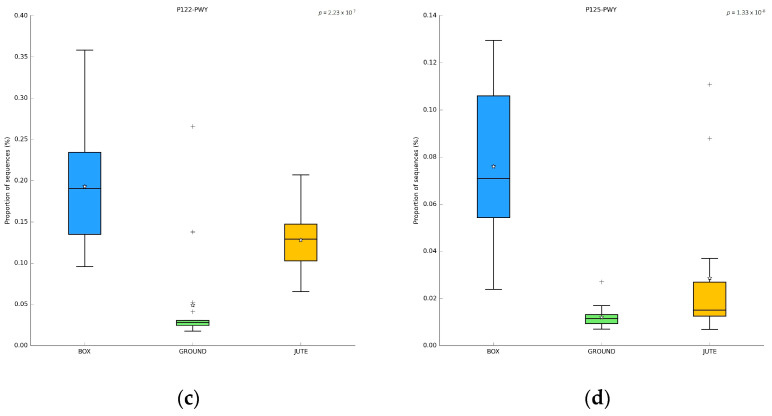
PICRUST analysis box plot: (**a**) 3-hydroxyphenyl-acetate degradation pathway; (**b**) degradation via β-ketoadipate pathway; (**c**) heterolactic fermentation; (**d**) 2,3-butanediol biosynthesis.

A PICRUST analysis was performed to predict the functional potential of microbial communities associated with fermented cocoa beans. The 3-hydroxyphenyl-acetate degradation pathway (Figure 8a) was classified as belonging to the group of aromatic compound degradation pathways, which includes pathways of different microorganisms that degrade substrates to serve as sources of nutrients and energy [27]. 4-Hydroxyphenyl-acetate is a typical aromatic amino acid fermentation product, but many bacteria convert it firstly into (3,4-dihydroxyphenyl) acetate, which is then degraded into TCA cycle intermediates, providing carbon and energy sources to the bacteria [49,50].

The aromatic compound degradation via β-ketoadipate (MetaCyc ID code PWY-5431 [27]), among the “Aromatic Compound Degradation” class, showed a significant difference between the BF method and GF method, in which the abundance was higher (Figure 8b). As reported by Caspi et al. (2018) [27], the expected taxonomic range was *Proteobacteria*, and *Pseudomonas putida* was highlighted among the species. In this study, *Pseudomonas* sp. was abundant in GF. The β-ketoadipate pathway is usually chromosomally encoded and, taking part in the degradation of naturally occurring aromatic compounds, is considered as a major utility pathway for the degradation of these aromatic compounds [51]. A variety of aromatic compounds, such as 4-hydroxybenzoate and benzoate, serve as growth substrates for bacteria through the β-ketoadipate pathway [51].

In addition, other bacteria employ this pathway, which involves two parallel branches, protocatechuate and catechol dissimilation [52]. Both these two pathways were significantly different for BF and GF, with a predominance for GF.

Protocatechuate is one of the intermediate metabolites in the microbial degradation of various compounds. Three pathways were identified: protocatechuate degradation I, protocatechuate degradation II (found in this study) and protocatechuate degradation III. The protocatechuate degradation II pathway, part of the β-ketoadipate pathway, is common among soil bacteria and fungi, differing in organization and regulation between the different species [51].

Catechol can be degraded in two ways: the *meta*-cleavage pathway and the *ortho*-cleavage pathway (found in this study, MetaCyc ID PWY-5417). The ortho-cleavage pathway employs the catechol 1,2-dioxygenase enzyme for catechol cleaving, and the result is further metabolized in 3-oxoadipate. This pathway is typical of soil bacteria and fungi, with differences in organization and regulation within the species [51,52,53,54]. The two phyla individuated by the MetaCyc database were *Actinobacteria* and *Proteobacteria*, with relevance in *Pseudomonas* [27].

Even if there are no data available for cocoa bean fermentation, in silico analysis suggests a clear difference between the BF and GF. Further research is needed to better evaluate the possible role of these pathways.

The P122-PWY pathway corresponds to heterolactic fermentation [27], and it is shown in Figure 8c. This fermentation significantly dominated samples from BF and JF, with very low abundance in GF samples. Homo- or hetero-fermentation converts glucose, still present after yeast growth, into lactic acid, acetic acid, carbon dioxide and/or ethanol. It was observed that the metabolic capabilities of LAB were most related to heterolactic fermentation and citrate assimilation pathways [55]. Fructose, still abundant, is fermented by fructose-favoring LAB species or is used as an alternative external electron acceptor by strictly heterofermentative LAB species, such as *L. fermentum* and *Weissella* strains. In the first 24 h, while yeast converts glucose into ethanol, citric acid is used as a co-substrate in the heterolactate fermentation, generating lactic acid or acetic acid and/or flavor compounds (diacetyl, acetoin and 2,3-butanediol) from pyruvate metabolism. This allows a slight increase in the pH of the pulp, with the relative proportion of all the end-product, enabling the modulation of firstly the composition of the cocoa fermenting mass and, consequently, the microbial succession and growth [3,12,30,44]. In particular, the slightly rising pH, due to the assimilation of citric acid by heterofermentative LAB, and the increase in oxygen, create the ideal condition for aerobic AAB growth [56]. In this way, there is a maximization of the LAB growth rate on glucose [1,46].

The P125-PWY corresponded to the 2,3-butanediol biosynthesis according to the MetaCyc database [27]. This pathway is part of the super-pathway of 2,3-butanediol biosynthesis (MetaCyc ID: PWY-6396), which includes several pathways found in different organisms, each producing various 2,3-butanediol stereoisomers. The super-pathway was observed mostly in BF samples as the P125-PWY shown in Figure 8d.

2,3-Butanediol could be fermented by many bacteria species, which could not carry the process all the way through, accumulating different intermediates, such as acetoin and diacetyl. Acetoin and diacetyl, a secondary metabolite of yeast and bacteria, are known for their characteristic taste of cream and butter [56,57,58]. Diacetyl was observed as an important flavor metabolite in dairy products, wine and beer [59]. On the other hand, acetoin, which is one of the desirable compounds for flavor development in cocoa bean fermentation, was observed to come from the metabolism of yeast, such as the two strains *S. cerevisiae* YB14 and *P. kudriavzevii* YP13 [60]. The 2,3-butanediol compound, produced by *Bacillus* spp., gives sweet and flowery notes during cocoa fermentation [4,56]. According to Visintin et al. (2017), Castro-Alayo et al. (2019) and Magalhães da Veiga Moreira et al. (2017) [57,60,61], the 2,3-butanediol compound was identified as a fine aroma component, detected in Brazil cocoa using two different starter mixes. Acetoin and 2,3-butanediol were found also after drying *Criollo* cocoa beans [60]. Diacetyl, 2,3-butanediol and acetoin, as well as acetaldehyde, were observed and found to increase sensory characteristics during fermentation [31].

Notably, L-leucine degradation and L-histidine degradation pathways were found to be statistically more abundant in GF than either BF or JF; this observation warrants further investigation.

### 3.7. Evaluation of the Three Fermentation Methods

Sufficient pulp drainage and avoiding over-dissipation of heat and moisture are two key points to result in a well-fermented mass [7,9]. Box fermentation, the most common method around the world [8], is preferred to ground fermentation, as it takes place in a more controllable environment and allows undesirable soil microbial contamination to be avoided. The microbial population of fermentation in jute bags, despite being widely used in Ecuadorian farms [9], is not as studied as ground and box populations, so further studies are necessary to better evaluate this fermentation method.

Both *Bacillus* sp. and *Tatumella* sp., found to bring potential positive characteristics to the finished fermented product [28,29,32,33,34,37], were detected exclusively in BF. Furthermore, *Hanseniaspora*, important for producing ethanol and secondary products [4,37], was one of the most relevant genera observed in BF. Moreover, the PICRUST analysis identified positive pathways in the BF, allowing the conclusion that fermentation in boxes is preferable.

In conclusion, it is important to clarify the differences between our studies and the literature. Some studies investigated the diversity in microorganisms during different spontaneous cocoa bean fermentations using mostly culture-dependent methods, which were intrinsically biased due to the impossibility of cultivating all the species. The coupling of classical methods with culture-independent molecular techniques could help in better detailing the microbial population [10,12,36,44,62]. Moreover, high-throughput sequencing (HTS) can be used to obtain greater resolution and detection sensitivity [63,64], even if it depends on DNA extraction methodologies and primer specificity, which could influence the richness and diversity of the microbial population investigated [14]. In addition, cocoa fermentation is still spontaneous and influenced by many factors, so differences, sometimes even very significant and unpredictable, can occur and have not been deeply studied yet. Therefore, further culture-dependent studies will reveal more precise aspects regarding microbial dynamics during the fermentations analyzed.

## 4. Conclusions

Analysis of the bacterial and fungal ecologies at different times of cocoa bean JF, GF and BF highlighted a strong variation in several species detected at the molecular level. A higher complexity was highlighted for the fungal population compared to LAB and AAB communities. The results indicated that the different fermentation methods modulate the adaptation of bacteria and fungi along the different days of fermentation investigated.

*L. fermentum* and *P. kudriavzevii* were the dominant microorganisms in cocoa bean fermentation and were found in BF, GR and JF.

*A. tropicalis*, *K. michiganensis* and *Fusarium solani*, *H. opuntiae*, *Pichia terricola* and *Candida ethanolica* dominated JF samples, resulting in a widely heterogeneous community. *Pseudomonas* sp., *S. cerevisiae* and *Hanseniaspora* sp. were the most abundant in GF. BF showed the dominance of *A. tropicalis* and *Bacillus* sp. for the bacterial community, but also *S. cerevisiae* and different species of *Hanseniaspora* (*H. uvarum*, *H. opuntiae* and *H. occidentalis*) among the fungal community.

The differences between days and the three fermentation methods highlighted the limited microbial variation and the presence of microorganisms that could guarantee a good fermentation in the box method.

PICRUST analysis was performed to identify potential interesting pathways. Differences between BF and GF methods were observed for the 3-hydroxyphenyl-acetate degradation pathway and degradation via the β-ketoadipate pathway, which prevailed in GF samples. On the other hand, heterolactic fermentation and 2,3-butanediol biosynthesis pathways were abundant in BF. To better understand the microbial community and its impact on the final chocolate quality, further studies should focus on microbial activities and their fermentation pathways.

## Data Availability

The datasets generated for this study are available on request to the corresponding author.
